# Evaluation of the Neonatal Sequential Organ Failure Assessment and Mortality Risk in Preterm Infants With Late-Onset Infection

**DOI:** 10.1001/jamanetworkopen.2020.36518

**Published:** 2021-02-04

**Authors:** Noa Fleiss, Sarah A. Coggins, Angela N. Lewis, Angela Zeigler, Krista E. Cooksey, L. Anne Walker, Ameena N. Husain, Brenda S. de Jong, Aaron Wallman-Stokes, Mhd Wael Alrifai, Douwe H. Visser, Misty Good, Brynne Sullivan, Richard A. Polin, Camilia R. Martin, James L. Wynn

**Affiliations:** 1 Department of Pediatrics, Columbia University School of Medicine, New York, New York; 2 Department of Pediatrics, Children’s Hospital of Philadelphia, Philadelphia, Pennsylvania; 3 Department of Pediatrics, Washington University School of Medicine, St Louis, Missouri; 4 Department of Pediatrics, University of Virginia School of Medicine, Charlottesville; 5 Department of Pediatrics, Vanderbilt University School of Medicine, Nashville, Tennessee; 6 Department of Neonatology, Amsterdam UMC University of Amsterdam, Vrije Universiteit, Emma Children’s Hospital, Amsterdam, the Netherlands; 7 Department of Pediatrics, Harvard Medical School, Boston, Massachusetts; 8 Department of Pediatrics, University of Florida School of Medicine, Gainesville

## Abstract

**Importance:**

Infection in neonates remains a substantial problem. Advances for this population are hindered by the absence of a consensus definition for sepsis. In adults, the Sequential Organ Failure Assessment (SOFA) operationalizes mortality risk with infection and defines sepsis. The generalizability of the neonatal SOFA (nSOFA) for neonatal late-onset infection-related mortality remains unknown.

**Objective:**

To determine the generalizability of the nSOFA for neonatal late-onset infection-related mortality across multiple sites.

**Design, Setting, and Participants:**

A multicenter retrospective cohort study was conducted at 7 academic neonatal intensive care units between January 1, 2010, and December 31, 2019. Participants included 653 preterm (<33 weeks) very low-birth-weight infants.

**Exposures:**

Late-onset (>72 hours of life) infection including bacteremia, fungemia, or surgical peritonitis.

**Main Outcomes and Measures:**

The primary outcome was late-onset infection episode mortality. The nSOFA scores from survivors and nonsurvivors with confirmed late-onset infection were compared at 9 time points (T) preceding and following event onset.

**Results:**

In the 653 infants who met inclusion criteria, median gestational age was 25.5 weeks (interquartile range, 24-27 weeks) and median birth weight was 780 g (interquartile range, 638-960 g). A total of 366 infants (56%) were male. Late-onset infection episode mortality occurred in 97 infants (15%). Area under the receiver operating characteristic curves for mortality in the total cohort ranged across study centers from 0.71 to 0.95 (T0 hours), 0.77 to 0.96 (T6 hours), and 0.78 to 0.96 (T12 hours), with utility noted at all centers and in aggregate. Using the maximum nSOFA score at T0 or T6, the area under the receiver operating characteristic curve for mortality was 0.88 (95% CI, 0.84-0.91). Analyses stratified by sex or Gram-stain identification of pathogen class or restricted to infants born at less than 25 weeks’ completed gestation did not reduce the association of the nSOFA score with infection-related mortality.

**Conclusions and Relevance:**

The nSOFA score was associated with late-onset infection mortality in preterm infants at the time of evaluation both in aggregate and in each center. These findings suggest that the nSOFA may serve as the foundation for a consensus definition of sepsis in this population.

## Key Points

**Question:**

How useful is the neonatal Sequential Organ Failure Assessment for identification of preterm infants at high risk for late-onset, infection-associated mortality?

**Findings:**

In this multicenter cohort study of 653 preterm infants with late-onset infection, the neonatal Sequential Organ Failure Assessment score was associated with infection-attributable mortality. Analyses stratified by sex or Gram stain of pathogen class or restricted to less than 25 weeks’ completed gestation did not reduce the association of the neonatal Sequential Organ Failure Assessment score with infection-related mortality.

**Meaning:**

In a large, multicenter cohort, the single-center–validated neonatal Sequential Organ Failure Assessment score was associated with mortality risk with late-onset infection in preterm infants, implying generalizability.

## Teaser

This cohort study assesses the generalizability of the neonatal Sequential Organ Failure Assessment metric for defining sepsis in neonates and estimating risk of infection-related mortality.

## Introduction

Although overall survival rates among preterm infants have improved, sepsis remains a significant factor associated with long-term morbidity and mortality in this population.^1,2,3,4^ Late-onset sepsis (sepsis that occurs after 72 hours of life during the birth hospitalization) affects 20% to 30% of the most preterm infants and has a 15% mortality rate.^5,6,7,8,9,10^ Despite multiple efforts, the methods of diagnosis, clinical management, and outcomes for preterm infants with late-onset sepsis have been largely unchanged over several decades. These findings are in part due to a lack of an accepted consensus definition for sepsis and a paucity of validated metrics reliably associated with sepsis-related mortality in this population.^11^

Consensus definitions are needed (1) for the development of standard research protocols, (2) to determine sample size, (3) to prevent misclassification of patients, and (4) to facilitate replication and improvement in future studies. Sepsis is defined as life-threatening organ dysfunction caused by a dysregulated host response to infection.^12^ Life-threatening organ dysfunction is substantiated by a Sequential Organ Failure Assessment (SOFA) score that categorically defines the extent of specific organ dysfunction that increases the risk of death or intensive care unit (ICU) admission.^12,13^ In contrast to adult and pediatric ICU environments, the distinction of infection from sepsis and the incorporation of organ dysfunction are not widely recognized in the neonatal ICU (NICU). Definitions of sepsis used in neonatology are mostly predicated on the isolation of bacterial pathogens from blood and/or the associated length of antimicrobial treatment.^5,7^ A failure to incorporate the presence or severity of organ dysfunction in preterm infants with late-onset sepsis prevents grouping of patients according to severity and mortality risk, which is necessary for advancements in research and improved outcomes.

The neonatal SOFA (nSOFA) was developed to address the need for a consensus definition for sepsis applicable to neonates.^14^ Similar to the adult SOFA and pediatric SOFA, the nSOFA serves as an operational definition of organ dysfunction that can identify those with a high risk of mortality among preterm infants with infection. Because there is no consensus definition for neonatal sepsis,^11^ the development of the nSOFA was informed by the progression of organ failure among preterm infants with lethal late-onset bacteremia and then validated in a single-center, retrospective cohort of preterm (<33 weeks) infants with late-onset bacteremia, fungemia, or intestinal perforation (included as an irrefutable source of infection).^14,15^ This validation study reported an association of higher nSOFA scores with infection-related mortality. With the goal of establishing the nSOFA score as a criterion for a consensus definition of sepsis in this population, in this study we determined the generalizability of the nSOFA for neonatal infection-related mortality risk in a multicenter, retrospective cohort of preterm (<33 weeks), very low-birth-weight (VLBW) infants with late-onset bacteremia, fungemia, or intestinal perforation.

## Methods

### Study Design

This multicenter retrospective cohort study measured the prognostic utility of the nSOFA for neonatal mortality among infants with late-onset infection (bacteremia, fungemia, or intestinal perforation) cared for in NICUs among 7 academic institutions: Columbia University Irving Medical Center, New York, New York; Beth Israel Deaconess Medical Center, Boston, Massachusetts; University of Florida, Gainesville; Vanderbilt University Medical Center, Nashville, Tennessee; Washington University, St Louis, Missouri; University of Virginia, Charlottesville; and Amsterdam University Medical Center, Amsterdam, the Netherlands. The study was conducted between January 1, 2010, and December 31, 2019. This study followed the Strengthening the Reporting of Observational Studies in Epidemiology (STROBE) reporting guideline for cohort studies. Each institution received approval for study participation with a waiver of informed consent from its institutional review board because the research was retrospective and data were deidentified; thus, the study involved no more than minimal risk to participants.

### Late-Onset Infection Episode

Because there is no consensus definition for neonatal sepsis,^11^ we considered a new bacteremic or fungemic event in an infant aged greater than 72 hours who received 5 or more days of antibiotic treatment or died before completing at least 5 days of treatment with intention to treat as a late-onset infection (LOI) episode. The onset of the LOI episode was defined as the time of clinician declaration of suspected infection, herein defined as the time of blood culture acquisition. Late-onset episodes of surgical peritonitis (perforated bowel associated with necrotizing enterocolitis or spontaneous intestinal perforation) with negative results of blood cultures were included as an irrefutable source of infection. Infants with positive blood cultures with organisms that are frequently considered contaminants, including *Bacillus* species and *Corynebacterium* species, were excluded.

### Inclusion and Exclusion Criteria

All sites used a standardized set of criteria to independently identify eligible events for inclusion. Inclusion criteria were new LOI (>72 hours of life) in VLBW, preterm (<33 weeks completed gestation) infants. Only the first episode of LOI was studied. An absence of antibiotic exposure for 48 hours before the LOI episode evaluation was required to facilitate characterization of an uninfected baseline in each patient. Each site abstracted and deidentified consecutive clinical and laboratory data from their electronic health records for all patients who met the inclusion criteria until a minimum of 5 patients who died with the LOI episode were identified. The frequency of LOIs as well as the availability of data at each center resulted in variation in the study period between centers, which overall ranged from 2010 to 2020. The primary outcome was episode mortality, defined as death that occurred during ongoing antibiotic therapy for an LOI episode. Birth weight, gestational age at birth, sex, age at event, pathogen identified, time from culture to death, and the suspected primary infection site were collected. Clinical data required to calculate the nSOFA score were collected and included the receipt of intubation and mechanical ventilation, the peripheral oxygen saturation, the fraction of inspired oxygen to achieve the peripheral oxygen saturation, the most current platelet count, and any requirement for glucocorticoid, inotropic, or vasoactive drugs.

### Application of the nSOFA Score

The nSOFA components and scoring paradigms were modeled after the adult SOFA.^12,13^ The nSOFA uses only objective and available clinical standard-of-care data to provide an operational definition of organ dysfunction that facilitates mortality risk stratification among VLBW infants with unequivocal LOI (bacteremia or intestinal perforation).^14^ The nSOFA uses categorical scores (total score range from 0 [best] to 15 [worst]) to objectively describe dynamic changes in (1) receipt of mechanical ventilation and oxygen to maintain a physiologic peripheral saturation (score range, 0-8); (2) inotropic or vasoactive drug support, including the use of corticosteroids for presumed adrenal insufficiency or catecholamine-resistant shock (score range, 0-4); and (3) the presence and severity of thrombocytopenia based on the most recent platelet measure (score range, 0-3) (Table 1; eAppendix in the Supplement).

**Table 1.** Neonatal Sequential Organ Failure Assessment (nSOFA) Components and Scoring^a^

Component	nSOFA Scores				
Respiratory score	0	2	4	6	8
Criteria	Not intubated or intubated, SpO_2_/Fio_2_≥300	Intubated, SpO_2_/Fio_2_<300	Intubated, SpO_2_/Fio_2_<200	Intubated, SpO_2_/Fio_2_<150	Intubated, SpO_2_/Fio_2_<100
Cardiovascular score	0	1	2	3	4
Criteria^b^	No inotropes and no systemic corticosteroid treatment	No inotropes and systemic corticosteroid treatment	1 inotrope and no systemic corticosteroid treatment	≥2 inotropes or 1 inotrope and systemic corticosteroid treatment	≥2 inotropes and systemic corticosteroid treatment
Hematologic score	0	1	2	3	NA
Criteria^c^	Platelet count ≥150 × 10^3^	Platelet count 100-149 × 10^3^	Platelet count <100 × 10^3^	Platelet count <50 × 10^3^	

Abbreviations: Fio_2_, fraction of inspired oxygen; NA, not applicable; SpO_2_, oxygen saturation as measured by pulse oxymetry.

SI conversion factor: To convert platelet count to ×10^9^/L, multiply by 1.

^a^ Score range, 0 (best) to 15 (worst).

^b^ Medications considered as inotropic or vasoactive included dopamine, dobutamine, epinephrine, norepinephrine, vasopressin, milrinone, and phenylephrine.

^c^ Most recent platelet count available to the clinician.

The nSOFA score was validated in an independent, single-center cohort of infants with LOI.^14^ In this multicenter, retrospective study to determine the generalizability of the nSOFA, each center identified qualifying infants and calculated the nSOFA score at 9 discrete time points, including and surrounding the LOI episode, using a web-based calculator and data from the electronic health records. The time the blood sample was drawn from the patient represented time 0 (T0). The time points that preceded (T minus 48 hours [T-48], T-24, T-12, and T-6) and followed (T plus 6 hours [T6], T12, T24, and T48) the infection evaluation were based on the progression of organ dysfunction in previous studies.^14,15^

### Statistical Analysis

Data were deidentified and analyzed in aggregate as well as by individual center. The nSOFA and demographic information were compared between infection survivors and nonsurvivors. For all data, tests of normality were performed before comparisons. For descriptive data comparisons, we used the Mann-Whitney test for nonparametric continuous data and the χ^2^ test or Fisher exact test for categorical data. Nonparametric continuous variables were summarized as medians with quartiles (25th and 75th percentiles). Categorical variables are presented as percentages. The threshold for statistical significance was *P* < .05 for 2-sided tests. Time point–based comparisons of nSOFA scores between designated groups were made using the Mann-Whitney test because the nSOFA score data were nonparametric. Area under the receiver operating characteristic curves (AUROCs) for mortality at designated points based on the nSOFA score were calculated using GraphPad Prism, version 8 (GraphPad Software) and the Wilson/Brown method. Chord diagrams were created to depict associations between organ dysfunction and mortality. Simple logistic regression generated estimated vs observed mortality. GraphPad Prism or R, version 3.6.2 (R Foundation for Statistical Computing) was used for all calculations.

## Results

### Patients

We identified 653 preterm VLBW infants with LOI with median gestational age 25.5 weeks (interquartile range [IQR], 24-27 weeks) and median birth weight 780 g (IQR, 638-960 g); 97 infants (15%) died with the LOI episode (Table 2; eFigure 1 in the Supplement). Of the 653 infants, 366 (56%) were male and 287 (44%) were female. Nonsurvivors were born at significantly lower gestational age than survivors (median, 25 weeks [IQR, 24-26 weeks] vs 26 weeks [IQR, 24-28 weeks]; *P* < .001) and infection occurred earlier after birth (median, 13 days [IQR, 7-26 days] vs 16 days [IQR, 9-32 days]; *P* = .04). Deaths considered to be associated with the LOI episode occurred in close proximity to the reference blood culture (median, 24 hours to death from reference blood culture; IQR, 11-78 hours). No statistically significant differences in sex or race were found between survivors and nonsurvivors. There were significant differences in organism distribution between survivors and nonsurvivors, with Gram-negative bacteremia occurring more frequently among nonsurvivors (46% [45 of 97] vs 19% [104 of 556]; *P* < .001) and gram-positive bacteremia occurring less frequently among nonsurvivors (31% [30 of 97] vs 70% [388 of 556]; *P* < .001). Infection site also differed significantly between survivors and nonsurvivors, with abdominal site infection (with or without a positive blood culture) occurring more frequently among nonsurvivors (46% [45 of 97] vs 18% [98 of 556]; *P* < .001). The 5 most commonly isolated bacteria from blood were coagulase-negative *Staphylococcus* species, *Staphylococcus aureus* (methicillin-sensitive), *Escherichia coli*, *Klebsiella pneumoniae*, and *Streptococcus agalactiae* (eTable 1 in the Supplement). The frequency of late mortality during the birth hospitalization among LOI episode survivors was 4% (23 of 556), occurred a median of 6.7 weeks after the reference culture (IQR, 3.3-11.6 weeks) at a median age of 9.3 weeks (IQR, 5.1-17.9 weeks), and was most common among infants born extremely preterm (median, 24 weeks; IQR, 24-27 weeks; mean [SD] birth weight, 677 [183] g) with severe bronchopulmonary dysplasia (14 of 23 [61%]).

**Table 2.** Demographic Characteristics of the Retrospective Cohort

Characteristic	No. (%)			*P* value
	All patients (n = 653)	Lived (n = 556)	Died (n = 97)	
Gestational age, median (IQR), wk	25.5 (24-27)	26.0 (24-28)	25.0 (24-26)	
≤24	224 (34)	176 (32)	48 (49)	<.001^a^
25-26	214 (33)	178 (32)	36 (37)	
27-28	126 (19)	118 (21)	8 (8)	
≥29	89 (14)	84 (15)	5 (5)	
Birth weight, median (IQR), g	780 (638-960)	800 (650-994)	680 (550-793)	
<750	283 (43)	220 (40)	63 (65)	<.001^a^
750-999	225 (34)	198 (36)	27 (28)	
≥1000	145 (22)	138 (25)	7 (7)	
Sex				
Male	366 (56)	310 (56)	56 (58)	.94^b^
Female	287 (44)	246 (44)	41 (42)	
Race				
Black	170 (26)	140 (25)	30 (31)	.63^c^
White	355 (54)	302 (54)	53 (55)	
Other	128 (20)	114 (21)	14 (14)	
Event day of life, median (IQR)	16 (9-31)	16 (9-32)	13 (7-26)	.04^a^
Pathogen^d^				
Gram-positive	418 (64)	388 (70)	30 (31)	<.001^c^
Gram-negative	149 (23)	104 (19)	45 (46)	
Fungus	15 (2)	13 (2)	2 (2)	
None identified	68 (10)	48 (9)	20 (21)	
Primary infection site				
Bacteremia/fungemia	336 (51)	303 (54)	33 (34)	<.001^c^
Abdomen	143 (22)	98 (18)	45 (46)	
CLABSI	33 (5)	26 (5)	7 (7)	
Other^e^	30 (5)	27 (5)	3 (3)	
Undetermined	111 (17)	102 (18)	9 (9)	

Abbreviations: CLABSI, central line–associated bloodstream infection; IQR, interquartile range.

^a^ Mann-Whitney test.

^b^ Fisher exact test.

^c^ χ^2^ test.

^d^ All patients had bacteremia or perforated bowel; 3 patients (all of whom survived) had both gram-positive and gram-negative organisms isolated from blood samples (not listed in the table).

^e^ Included pulmonary, renal, central nervous system, and soft-tissue sites.

### nSOFA Comparisons

**Figure 1.**
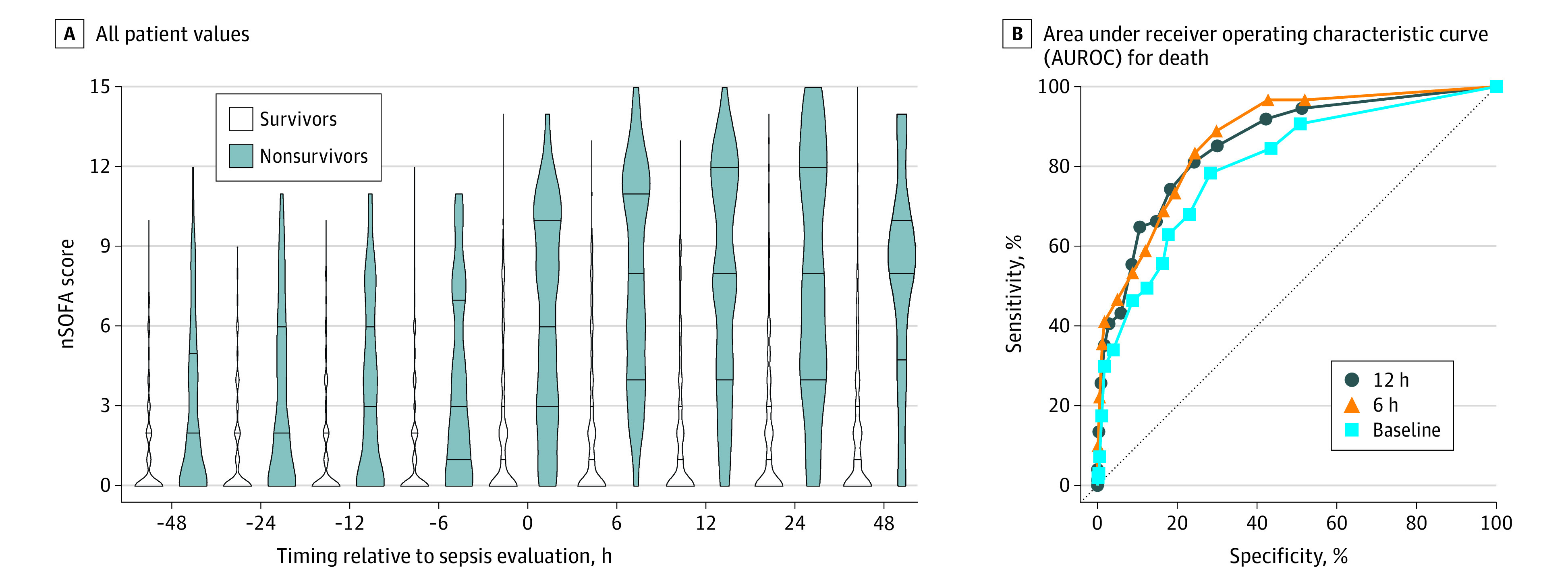
Neonatal Sequential Organ Failure Assessment (nSOFA) Scores Among Preterm Survivors and Nonsurvivors With Late-Onset Infection A, All values for survivors (n = 556) and nonsurvivors (n = 97). Violin plots represent group medians at each time point. Error bars represent interquartile ranges. B, Area under receiver operating characteristic curve (AUROC) for death shown for T0 (0.81; 95% CI, 0.76-0.85), T6 (0.87; 95% CI, 0.83-0.91), and T12 (0.86; 95% CI, 0.81-0.91) time points. All AUROCs: *P* < .001.

**Figure 2.**
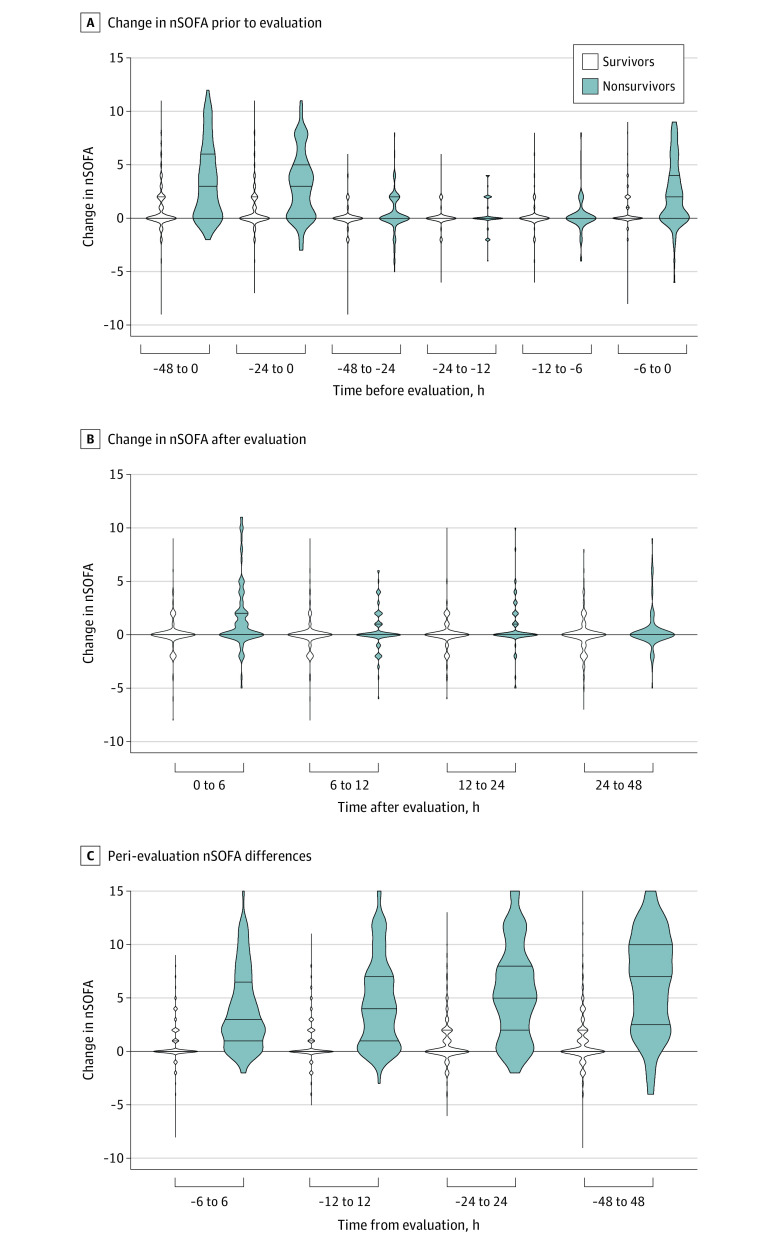
Change in Neonatal Sequential Organ Failure Assessment (nSOFA) Score With an Infection Episode Among Survivors and Nonsurvivors All values for survivors (n = 556) and nonsurvivors (n = 97). Violin plots represent group medians at each time point. Error bars represent interquartile ranges (IQRs). A, Before evaluation, the change in the nSOFA score was most pronounced among nonsurvivors between T-48 and T0 (median, 3; IQR, 0-6), T-24 and T0 (median, 3; IQR, 0-5), and T-6 and T0 (median, 2; IQR, 0-4); all *P* < .001. B, Change in the nSOFA score at postevaluation time points. C, Peri-evaluation nSOFA score differences increased proportionally to the length of time interval measured (T-6 to T6: median, 3 (IQR, 1-7); T-12 to T12: median, 4 (IQR, 1-7); T-24 to T24: median, 5 (IQR, 2-8); and T-48 to T48: median, 7 (IQR, 3-10). For patients who died before a time point measured, the most recent nSOFA score was used for calculations.

Compared with survivors, nonsurvivors had elevated total and individual component nSOFA scores at all time points evaluated (Figure 1; eFigure 2 in the Supplement). The AUROC for mortality in the total cohort measured at T0 was 0.81 (95% CI, 0.76-0.85); T6, 0.87 (95% CI, 0.83-0.91); and T12, 0.86 (95% CI, 0.81-0.91) (Figure 1). Center-specific AUROC ranges were 0.71 to 0.95 (T0), 0.77 to 0.96 (T6), and 0.78 to 0.96 (T12), with utility noted at all centers (eTable 2 in the Supplement). Using the maximum nSOFA score at T0 or T6, the AUROC for mortality was 0.88 (95% CI, 0.84-0.91); logistic regression identified associations between higher T0 and T6 nSOFA scores and death (eFigure 3 in the Supplement). The mortality rate among patients with a maximum nSOFA score at T0 or T6 less than 4 was 2.4% (10 of 423), whereas mortality occurred in 81% of infants with maximum nSOFA scores at T0 or T6 of greater than or equal to 10. The likelihood ratio for mortality progressively increased as the nSOFA score increased (2-fold with nSOFA score ≥2, 4-fold with score ≥6, 8-fold with score ≥8, and 16-fold with score ≥10) (eFigure 4 in the Supplement). Mortality risk increased with nSOFA score category at all time points analyzed (eTable 3 in the Supplement). Because a large portion of LOI episode-related mortality occurred in the most preterm infants, we performed a subanalysis on infants who had completed less than 25 weeks’ gestation at birth (n = 224). At all preinfection time points, nSOFA scores were higher among survivors less than 25 weeks compared with those greater than or equal to 25 weeks (median 2 [IQR, 0-4] v 0 [IQR, 0-1]; all *P* < .001) (eFigure 5 in the Supplement). Similarly, median nSOFA scores were higher among nonsurvivors less than 25 weeks compared with those greater than or equal to 25 weeks (T-48, 3 [IQR, 0-6] vs 1 [IQR, 0-4]; T-24, 4 [IQR, 1-8] vs 1 [IQR, 0-4]; T-12, 5 [IQR, 1-8] vs 2 [IQR, 0-4]; T-6, 4 [IQR, 2-8] v 2 [IQR, 0-5]; all *P* < .05) (eFigure 5 in the Supplement).Differences in nSOFA scores between infants born at less than 25 weeks of completed gestation who were survivors and nonsurvivors were present at all time points between groups. AUROCs for mortality in infants born at less than 25 weeks of gestation were also comparable with results in the total cohort: T0, 0.78 (95% CI, 0.70-0.86); T6, 0.83 (95% CI, 0.76-0.89); and T12, 0.83 (95% CI, 0.77-0.90). The change in nSOFA scores was most pronounced among nonsurvivors between T-48 and T0 (median, 3; IQR, 0-6), T-24 and T0 (median, 3; IQR, 0-5), and T-6 and T0 (median, 2; IQR, 0-4) (all *P* < .001) (Figure 2; eFigure 6 and eFigure 7 in the Supplement). Comparable nSOFA score changes occurred in infants born at less than 25 weeks’ gestation (eFigure 8 in the Supplement).

Nonsurvivors showed baseline differences as well as a progressive increase in the frequency of respiratory, cardiovascular, and hematologic dysfunction compared with survivors (eFigure 2 in the Supplement). Respiratory dysfunction divergence was present as early as T-12. Compared with survivors, nonsurvivors manifested the greatest divergence in cardiovascular component scores at T6. Hematologic dysfunction divergence between nonsurvivors and survivors was most pronounced at T0 and was sustained through T48. Chord diagrams summarize the frequency and extent of organ dysfunction between survivors and nonsurvivors at each of the 9 time points examined (eFigure 9 in the Supplement).

When survivors and nonsurvivors were compared by sex, no significant differences between groups at any time point were present (eFigure 10 in the Supplement). Similarly, a comparison of survivors and nonsurvivors by Gram-stain class isolated from blood revealed comparable trends in nSOFA with results in the total cohort (eFigure 11 in the Supplement). nSOFA profiles among infants with a perforated bowel but negative blood cultures showed analogous changes between survivors and nonsurvivors with results in the total cohort (eFigure 12 in the Supplement). The frequency of late mortality during the birth hospitalization among LOI survivors in this multicenter study was 4% (23 of 556; median, 6.7 weeks after the reference culture; IQR, 3.3-11.6 weeks; median age, 9.3 weeks; IQR, 5.1-17.9 weeks) and was most common among infants born extremely preterm (median, 24 weeks; IQR, 24-27 weeks; mean [SD] birth weight, 677 [183] g) with severe bronchopulmonary dysplasia (14 of 23 [61%]).

## Discussion

In adults with suspected infection, the definition of sepsis is based on the presence of life-threatening organ dysfunction, reflected in the SOFA score, which is associated with ICU admission or mortality.^12^ To reach a consensus definition of sepsis in the neonatal population, it is essential to establish an operational definition of life-threatening organ dysfunction applicable to the preterm population (nSOFA). Because preterm neonates are not often cared for outside of the NICU, the most severe outcome (mortality) among those with an infection must be used. The nSOFA, an adaptation of the SOFA specific to neonates, demonstrated generalizability in aggregate and in each center as a risk assessment tool that identified LOI-associated mortality risk among VLBW infants in this multi-center cohort study. Taken together, the nSOFA score may serve as a useful criterion on which to build a consensus definition of sepsis in this population.

A lack of developmental age-specific criteria for life-threatening organ dysfunction applicable to the preterm population prevents meaningful classification and study of infection as well as other clinical entities in this population. Infants at the lowest extremes of gestational age are at disproportionately higher risk for late-onset infection owing to physiologic immaturity as well as critical illness that requires prolonged NICU hospitalizations.^16,17^ However, the nSOFA demonstrated consistent associations with LOI-associated mortality even in the lowest gestational age strata. Stratified analyses by sex showed negligible differences in the nSOFA performance. Similarly, the analyses stratified by Gram-stain class of the isolated pathogen showed similar extents of organ dysfunction among LOI survivors and nonsurvivors.

We identified several relevant parallels of our study with studies on SOFA performance. An increase in the SOFA score over time (≥2 points) was predictive of mortality risk.^18^ A systematic review and meta-regression of SOFA as a predictor of mortality in adults found the change in SOFA score from baseline to be the metric most reliably associated with mortality^19^; this finding was reflected in our study of preterm infants, in which a 2-point increase in the nSOFA score was associated with a 2-fold increase in the odds of mortality. Similarly, organ dysfunction scores are widely used as metrics associated with mortality as well as clinical course in pediatric and adult ICU populations and have been validated across multiple disease states.^20,21,22,23,24,25,26^ No risk of mortality, clinical course description, or severity of illness scoring paradigms are routinely used in neonatal critical care. Although life-threatening organ dysfunction is a requirement to distinguish sepsis from infection, organ dysfunction accompanies multiple disease states that commonly occur in the NICU. As an objective measure of clinical course, the nSOFA has potential utility as both a surrogate variable for illness severity and a metric for risk assessment of short- and long-term clinical end points.

We have developed an international consortium of academic centers that have integrated the nSOFA into their electronic health records for all NICU patients. This consortium may enable nSOFA-focused prospective studies, as well as quality improvement initiatives, aimed at short- and long-term outcomes in the NICU. We do not advocate for use of the nSOFA in clinical practice at this time. Rather, these data are evidence that the nSOFA can be used to classify mortality risk among preterm infants with suspected infection for future studies. A precision medicine approach that uses patient-specific stratification of infection mortality risk is needed to establish useful diagnostic testing that will both facilitate effective antibiotic stewardship and identify those that are most likely to benefit from potential adjunctive treatments.

### Limitations

This study has limitations. The nSOFA scores may have been influenced by institution-specific approaches to care, including variations in thresholds for intubation, corticosteroid or vasopressor use, and laboratory monitoring. Site-specific analyses revealed negligible effects of intersite variations across 9 time points in a 96-hour window surrounding the episode. It was not possible to determine with certainty that the cause of death was secondary to infection alone. To mitigate this potential limitation, we studied only patients with documented bacteremia, fungemia, or a perforated bowel, and all deaths considered infection-related occurred in close proximity to the reference culture and while receiving antibiotics for the infection episode evaluated. Taken together, these attributes suggest infection was a significant contributor to mortality. We could not include a focus on meningitis because of a limited number of documented cases (n = 3), consistent with previous reports in this population indicating that meningitis is rare and frequently associated with bacteremia.^27^ In contrast to adults and children, who may experience high rates of late mortality (after antimicrobial treatment is completed) following sepsis, the frequency of late mortality during the birth hospitalization among LOI episode survivors in this multicenter study was low, it occurred several weeks after the reference culture, and it was most common among infants born extremely preterm with severe bronchopulmonary dysplasia. It could not be determined whether the cause of late mortality was primarily the result of complications of extreme prematurity, the impact of the previous infection, or a combination of factors. These questions may be answered via study of larger cohorts. This was a study to assess the generalizability of the nSOFA score in its current iteration. We concede that the score can always be improved as more data become available. We acknowledge the potential limitation that changes in demographics and standards of care may have occurred over the study period.

## Conclusions

Among preterm VLBW infants with confirmed LOI episode, the nSOFA showed generalizable utility as an operational definition of organ dysfunction associated with mortality risk unaffected by sex, pathogen, specific center, or extreme prematurity. The nSOFA may provide the requisite foundation on which to build a consensus definition for sepsis in preterm neonates.

## Notes

Supplement. **eFigure 1.** Cohort Flow Diagram **eTable 1.** Microbiologic Isolates From Blood **eFigure 2.** nSOFA Component Profiles for Survivors and Nonsurvivors **eTable 2.** nSOFA Prognostic Utility for Mortality at Single Centers **eFigure 3.** Utility of the Maximum nSOFA Score at Either T0 or T6 **eTable 3.** nSOFA Scores by Timepoint Among Survivors and Nonsurvivors by Scoring Group **eFigure 4.** Likelihood Ratios for Mortality by nSOFA Score Cutoff at T0, T6, T12 and for the nSOFA^max^ T0, T6 **eFigure 5.** Performance of nSOFA Among Infants <25 Weeks **eFigure 6.** Temporal Progression of nSOFA Scores Among Survivors and Nonsurvivors **eFigure 7.** Temporal Progression of Individual Patient nSOFA**^Δ^** Dynamics Among Survivors and Nonsurvivors **eFigure 8.** Individual Patient-Level Organ Dysfunction Dynamics Among <25 Weeks GA Survivors and Nonsurvivors **eFigure 9.** Prevalence and Temporal Progression of Organ Dysfunction Between Survivors and Nonsurvivors **eFigure 10.** nSOFA Scores for Females and Males **eFigure 11.** nSOFA Scores by Pathogen Classification **eFigure 12.** nSOFA Scores Among Patients With Surgical Peritonitis and Negative Blood Cultures **eAppendix.** Neonatal Sequential Organ Failure Assessment (nSOFA) **eReferences**
