# Supplementary material for: Evaluation of the Neonatal Sequential Organ Failure Assessment and Mortality Risk in Preterm Infants With Late-Onset Infection
Source: JAMA Netw Open. 2021 Feb 4;4(2):e2036518. doi: 10.1001/jamanetworkopen.2020.36518 (PMC7862993; doi:10.1001/jamanetworkopen.2020.36518)
Supplement: Supplement. — eFigure 1. Cohort Flow Diagram eTable 1. Microbiologic Isolates From Blood eFigure 2. nSOFA Component Profiles for Survivors and Nonsurvivors eTable 2. nSOFA Prognostic Utility for Mortality at Single Centers eFigure 3. Utility of the Maximum nSOFA Score at Either T0 or T6 eTable 3. nSOFA Scores by Timepoint Among Survivors and Nonsurvivors by Scoring Group eFigure 4. Likelihood Ratios for Mortality by nSOFA Score Cutoff at T0, T6, T12 and for the nSOFAmax T0, T6 eFigure 5. Performance of nSOFA Among Infants <25 Weeks eFigure 6. Temporal Progression of nSOFA Scores Among Survivors and Nonsurvivors eFigure 7. Temporal Progression of Individual Patient nSOFAΔ Dynamics Among Survivors and Nonsurvivors eFigure 8. Individual Patient-Level Organ Dysfunction Dynamics Among <25 Weeks GA Survivors and Nonsurvivors eFigure 9. Prevalence and Temporal Progression of Organ Dysfunction Between Survivors and Nonsurvivors eFigure 10. nSOFA Scores for Females and Males eFigure 11. nSOFA Scores by Pathogen Classification eFigure 12. nSOFA Scores Among Patients With Surgical Peritonitis and Negative Blood Cultures eAppendix. Neonatal Sequential Organ Failure Assessment (nSOFA) eReferences [file jamanetwopen-e2036518-s001.pdf]

Supplementary Online Content

Fleiss N, Coggins SA, Lewis AN, et al. Evaluation of the neonatal sequential organ failure assessment and mortality risk in preterm infants with late-onset infection. *JAMA Netw Open*. 2021;4(2):e2036518. doi:10.1001/jamanetworkopen.2020.36518

eFigure 1. Cohort Flow Diagram

eTable 1. Microbiologic Isolates From Blood

eFigure 2. nSOFA Component Profiles for Survivors and Nonsurvivors

eTable 2. nSOFA Prognostic Utility for Mortality at Single Centers

eFigure 3. Utility of the Maximum nSOFA Score at Either T0 or T6

eTable 3. nSOFA Scores by Timepoint Among Survivors and Nonsurvivors by Scoring Group

eFigure 4. Likelihood Ratios for Mortality by nSOFA Score Cutoff at T0, T6, T12 and for the $nSOFA^{max}$ T0, T6

eFigure 5. Performance of nSOFA Among Infants <25 Weeks

eFigure 6. Temporal Progression of nSOFA Scores Among Survivors and Nonsurvivors

eFigure 7. Temporal Progression of Individual Patient $nSOFA^{\Delta}$ Dynamics Among Survivors and Nonsurvivors

eFigure 8. Individual Patient-Level Organ Dysfunction Dynamics Among <25 Weeks GA Survivors and Nonsurvivors

eFigure 9. Prevalence and Temporal Progression of Organ Dysfunction Between Survivors and Nonsurvivors

eFigure 10. nSOFA Scores for Females and Males

eFigure 11. nSOFA Scores by Pathogen Classification

eFigure 12. nSOFA Scores Among Patients With Surgical Peritonitis and Negative Blood Cultures

eAppendix. Neonatal Sequential Organ Failure Assessment (nSOFA)

eReferences

This supplementary material has been provided by the authors to give readers additional information about their work.

© 2021 Fleiss N et al. *JAMA Network Open.*

eFigure 1: Cohort flow diagram

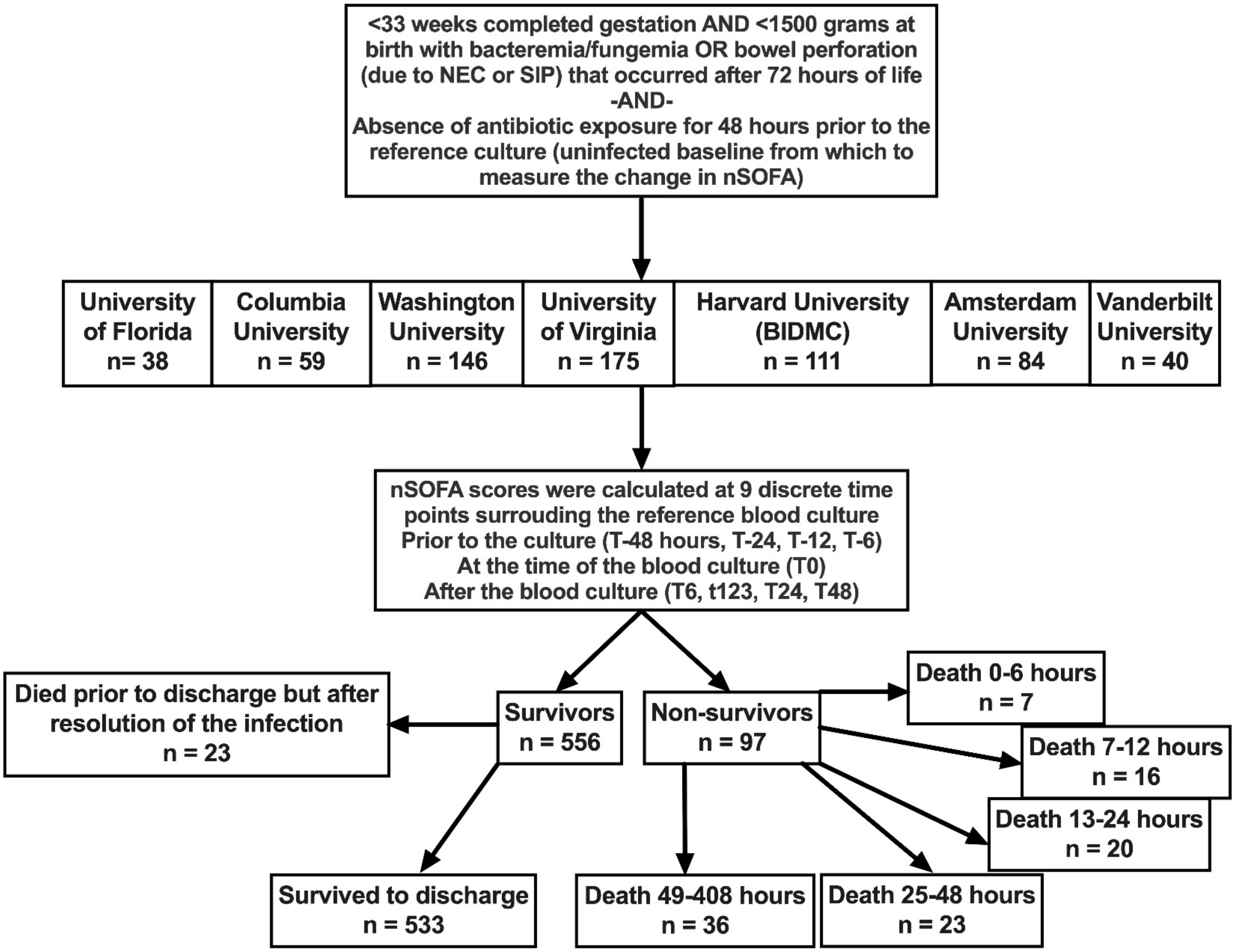

© 2021 Fleiss N et al. *JAMA Network Open.*

eTable 1. Microbiologic isolates from blood

	All	Lived (n)	Died (n, %)
**Gram positive**	**418**	**388**	**30 (7.2)**
Coagulase-negative Staphylococcus	258	235	23 (8.9)
Staphylococcus aureus, methicillin sensitive	87	84	3 (3.4)
Streptococcus agalactiae	28	26	2 (7.1)
Staphylococcus aureus, methicillin resistant	19	19	0
Enterococcus faecalis	16	15	1 (6.3)
Streptococcus spp	7	7	0
Clostridium perfringens	2	1	1 (50)
Propionibacterium acnes	1	1	0
**Gram negative**	**149**	**104**	**45** (30.2)
Escherichia coli	57	41	16 (28.1)
Klebsiella pneumoniae	39	29	10 (25.6)
Pseudomonas aeruginosa	15	4	11 (73.3)
Serratia marcescens	15	12	3 (20)
Enterobacter cloacae	10	10	0
Enterobacter aerogenes	4	2	2 (50)
Citrobacter spp	3	1	2 (66.7)
Klebsiella oxytoca	3	2	1 (33.3)
Rauoltella spp	2	2	0
Prevotella bivia	1	1	0
**Fungus**	**15**	**13**	**2** (13.3)
Candida albicans	10	9	1 (10)
Candida spp	5	4	1 (20)

Three patients had both Gram positive and Gram negative organisms isolated from blood (not listed in this table); all survived.
Sixty-eight patients experienced bowel perforation without organism isolation from blood; 20 died.

© 2021 Fleiss N et al. *JAMA Network Open.*

eFigure 2. nSOFA component profiles for survivors and nonsurvivors. All patient values (survivors and non-survivors) are shown. Violin plots represent group medians at each time point. Error bars represent interquartile ranges (IQR). **A.** Respiratory component. **B.** Cardiovascular component. **C.** Hematologic component. Comparisons by Mann-Whitney. * designates p-value <0.001.

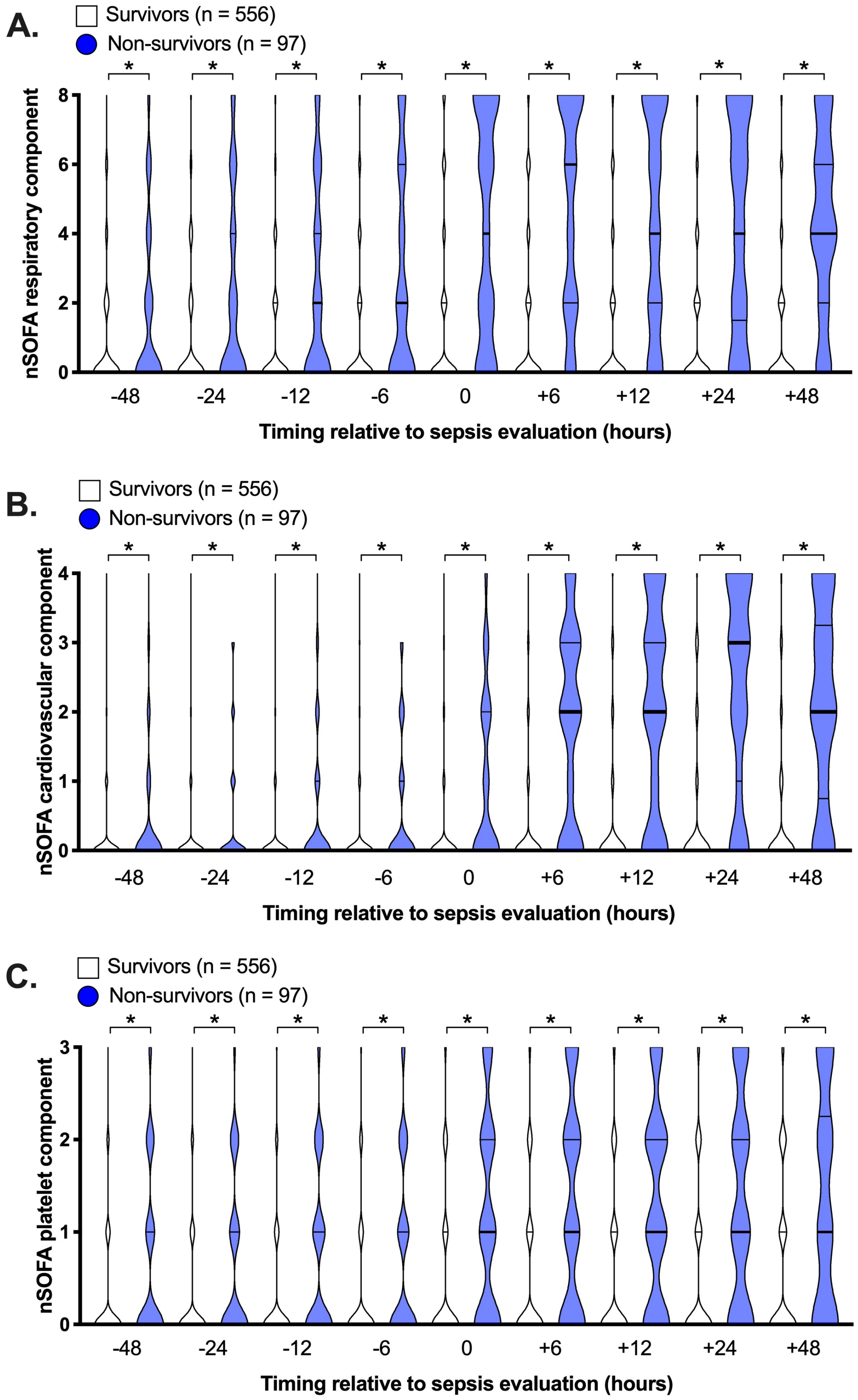

© 2021 Fleiss N et al. *JAMA Network Open.*

eTable 2. nSOFA prognostic utility for mortality at single centers.

Center	AUROC	95% CI	p value
**University of Florida, 2016-2018 (31 survivors; 7 non-survivors)**			
T0	0.9516	0.8868 to 1.000	<0.001
T6	0.9562	0.8842 to 1.000	<0.001
T12	0.9562	0.8746 to 1.000	<0.001
**Columbia University (Irving), 2013-2018 (53 survivors; 6 non-survivors)**			
T0	0.9403	0.8599 to 1.000	<0.001
T6	0.9560	0.8774 to 1.000	<0.001
T12	0.9088	0.7524 to 1.000	0.02
**Amsterdam University, 2017-2018 (67 survivors; 17 non-survivors)**			
T0	0.7103	0.5499 to 0.8707	0.008
T6	0.8705	0.7672 to 0.9738	<0.001
T12	0.7904	0.6143 to 0.9664	0.002
**Beth Israel Deaconess, Harvard University, 2010-2019 (102 survivors; 9 non-survivors)**			
T0	0.8170	0.6435 to 0.9905	0.002
T6	0.8897	0.7342 to 1.000	<0.001
T12	0.8235	0.5935 to 1.000	0.01
**Washington University, 2015-2019 (114 survivors; 32 non-survivors)**			
T0	0.7133	0.6088 to 0.8177	<0.001
T6	0.7691	0.6791 to 0.8591	<0.001
T12	0.7772	0.6835 to 0.8708	<0.001
**University of Virginia, 2011-2019 (162 survivors; 13 non-survivors)**			
T0	0.8872	0.8145 to 0.9600	<0.001
T6	0.9318	0.8827 to 0.9810	<0.001
T12	0.9405	0.8789 to 1.000	<0.001
**Vanderbilt University, 2017-2019 (27 survivors; 13 non-survivors)**			
T0	0.7293	0.5694 to 0.8893	0.02
T6	0.7660	0.6121 to 0.9199	0.01
T12	0.8316	0.7051 to 0.9582	0.002

© 2021 Fleiss N et al. *JAMA Network Open.*

eFigure 3. Utility of the maximum nSOFA score at either T0 or T6 ($nSOFA^{max}$ T0, T6). A. Violin plot of the $nSOFA^{max}$ T0, T6 for survivors and non-survivors. Black bars represent the median and interquartile range. Comparison by Mann-Whitney. **B.** Area under receiver operating characteristics curve (AUROC) for death, p<0.001. **C.** Predicted (red band with 95% confidence intervals) versus observed (solid line) mortality using the $nSOFA^{max}$ T0, T6. * designates p-value <0.001.

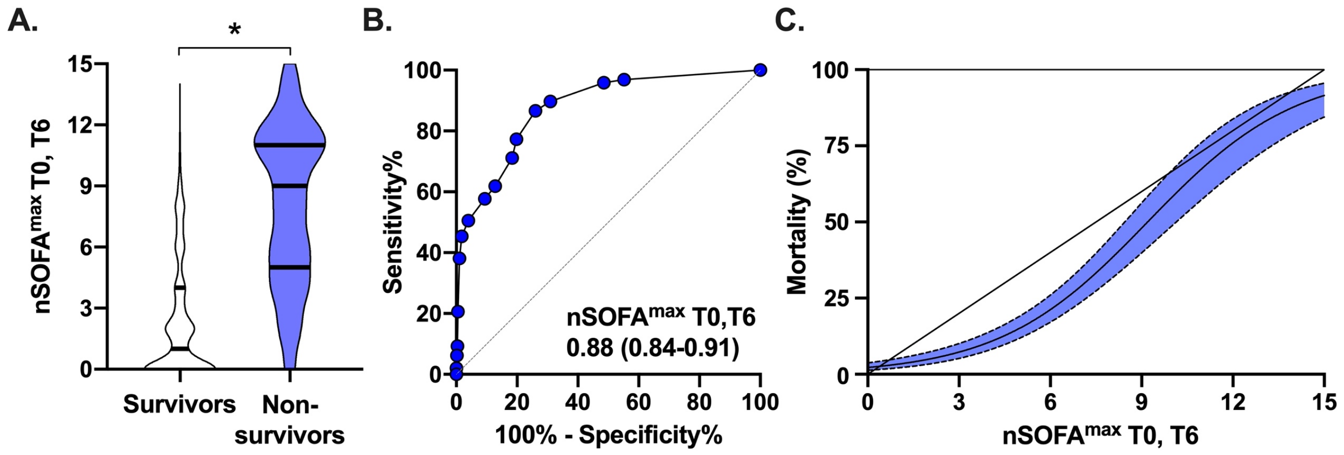

© 2021 Fleiss N et al. *JAMA Network Open.*

eTable 3. nSOFA scores by timepoint among survivors and non-survivors by scoring group.

	nSOFA score						
	**0-3**	**4-7**	**8-11**	**≥12**	**Deaths (during interval)**		**Deaths (cumulative)**
T0 lived (n)	428	79	46	3	NA		NA
T0 died (n)	31	21	38	7	NA		NA
T6 lived (n)	420	87	46	3	NA		NA
T6 died (n)	15	27	28	20	7		7
T12 lived (n)	421	87	43	5	NA		NA
T12 died (n)	14	19	22	19	16		23
T24 lived (n)	421	97	29	9	NA		NA
T24 died (n)	10	16	12	16	20		43
T48 lived (n)	420	100	28	8	NA		NA
T48 died (n)	5	7	17	5	23		63

© 2021 Fleiss N et al. *JAMA Network Open.*

eFigure 4. Likelihood ratios for mortality by nSOFA score cutoff at T0, T6, T12 and for the nSOFA^{max} T0, T6. The mortality rate among patients with an nSOFA^{max} at T0 or T6 of <4 was 2.4%, whereas mortality occurred in 81% with nSOFA^{max} at T0 or T6 of $\geq$10. The likelihood ratio for mortality progressively increased as the nSOFA increased (2-fold with nSOFA of $\geq$2, 4-fold with $\geq$6, 8-fold with $\geq$8, 16-fold with $\geq$10.

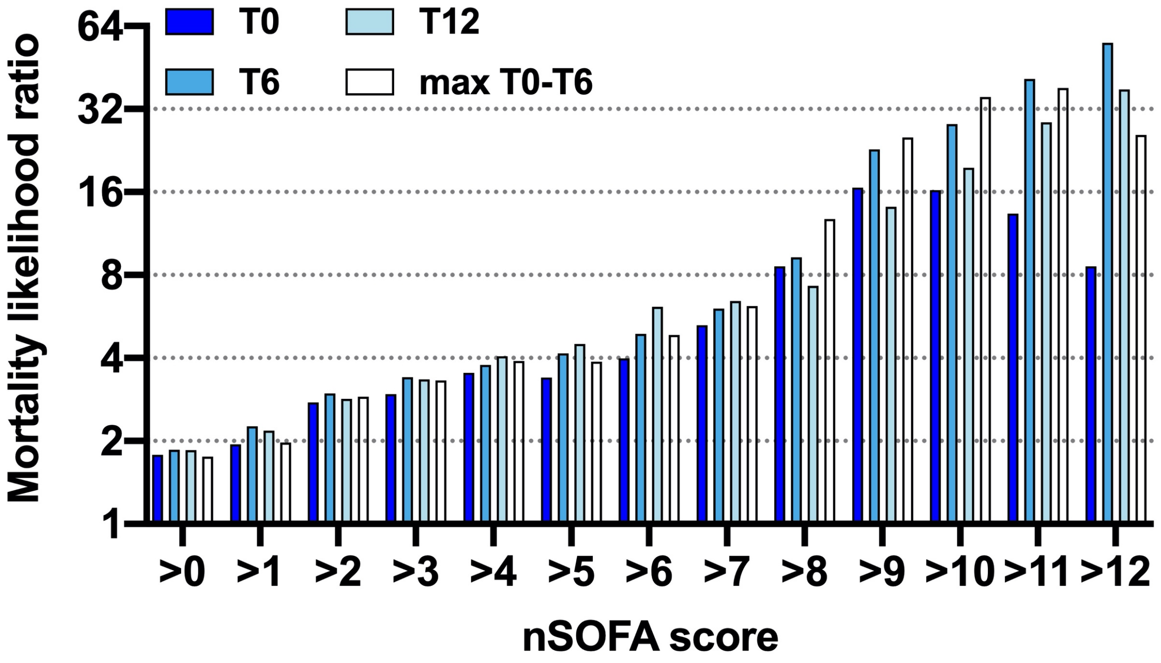

© 2021 Fleiss N et al. *JAMA Network Open.*

eFigure 5. Performance of nSOFA among infants <25 weeks.

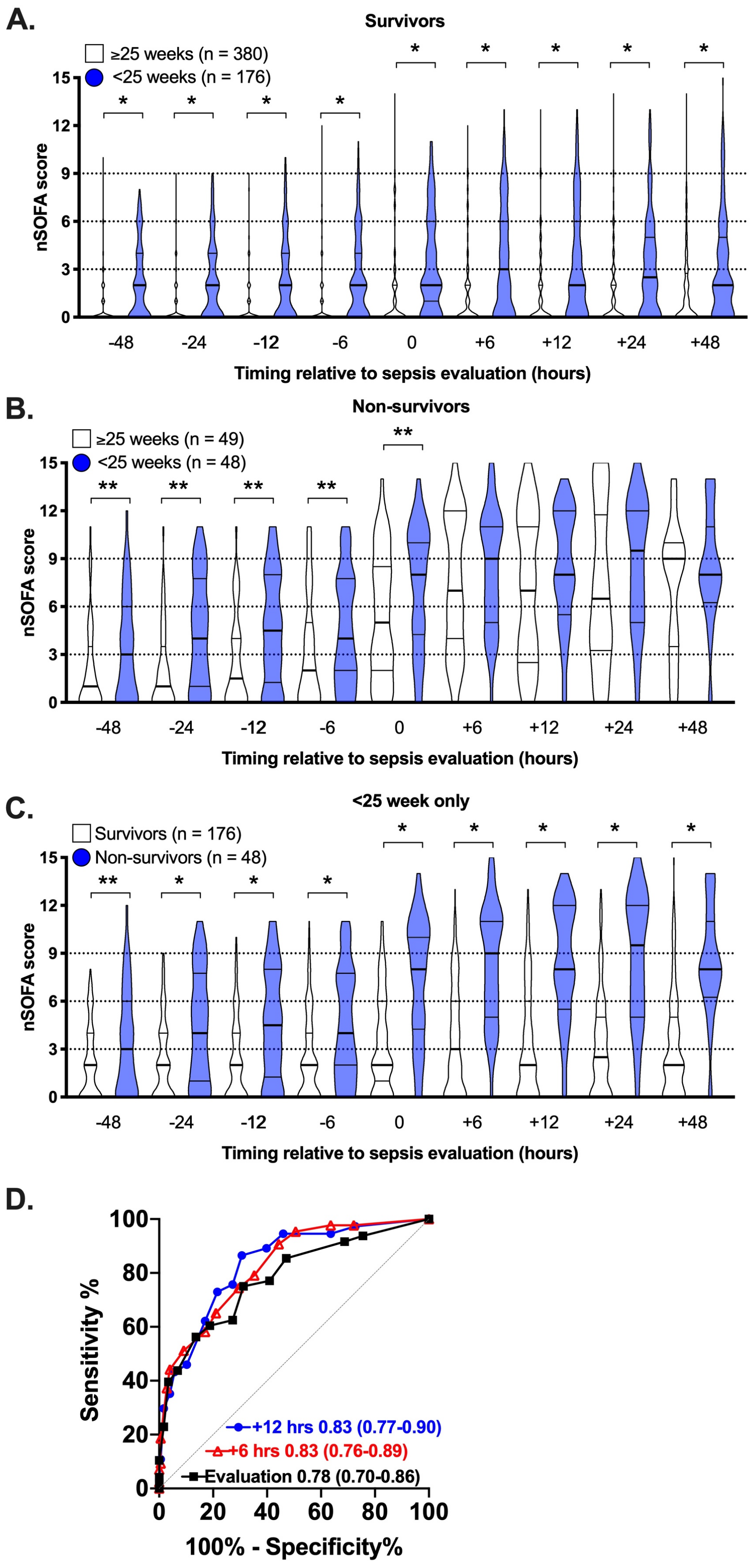

© 2021 Fleiss N et al. *JAMA Network Open.*

eFigure 5 continued. All patient values (survivors and non-survivors) are shown. Violin plots represent group medians at each time point. Error bars represent interquartile ranges (IQR). **A.** Comparison of nSOFA scores among survivors <25 weeks and ≥25 weeks. **B.** Comparison of nSOFA scores among non-survivors <25 weeks and ≥25 weeks. **C.** nSOFA scores among <25 weeks GA survivors and non-survivors with late-onset infection. **D.** Area under receiver operating characteristics curves for the nSOFA to predict mortality in <25 week infants at T0, T6, and T12 time points (all p<0.001). Comparisons by Mann-Whitney. * designates p-value <0.001. ** designates p-value <0.05.

© 2021 Fleiss N et al. *JAMA Network Open.*

eFigure 6. Temporal progression of nSOFA scores among survivors and non-survivors. All patient values (survivors and non-survivors) are shown. Each row in the heat map represents a single patient. Each column represents a single time point. Patients in each group (survivors and non-survivors) are sorted by descending T0 nSOFA scores. The cell color and intensity reflects the nSOFA score (legend). Black cells indicate mortality prior to the column time point.

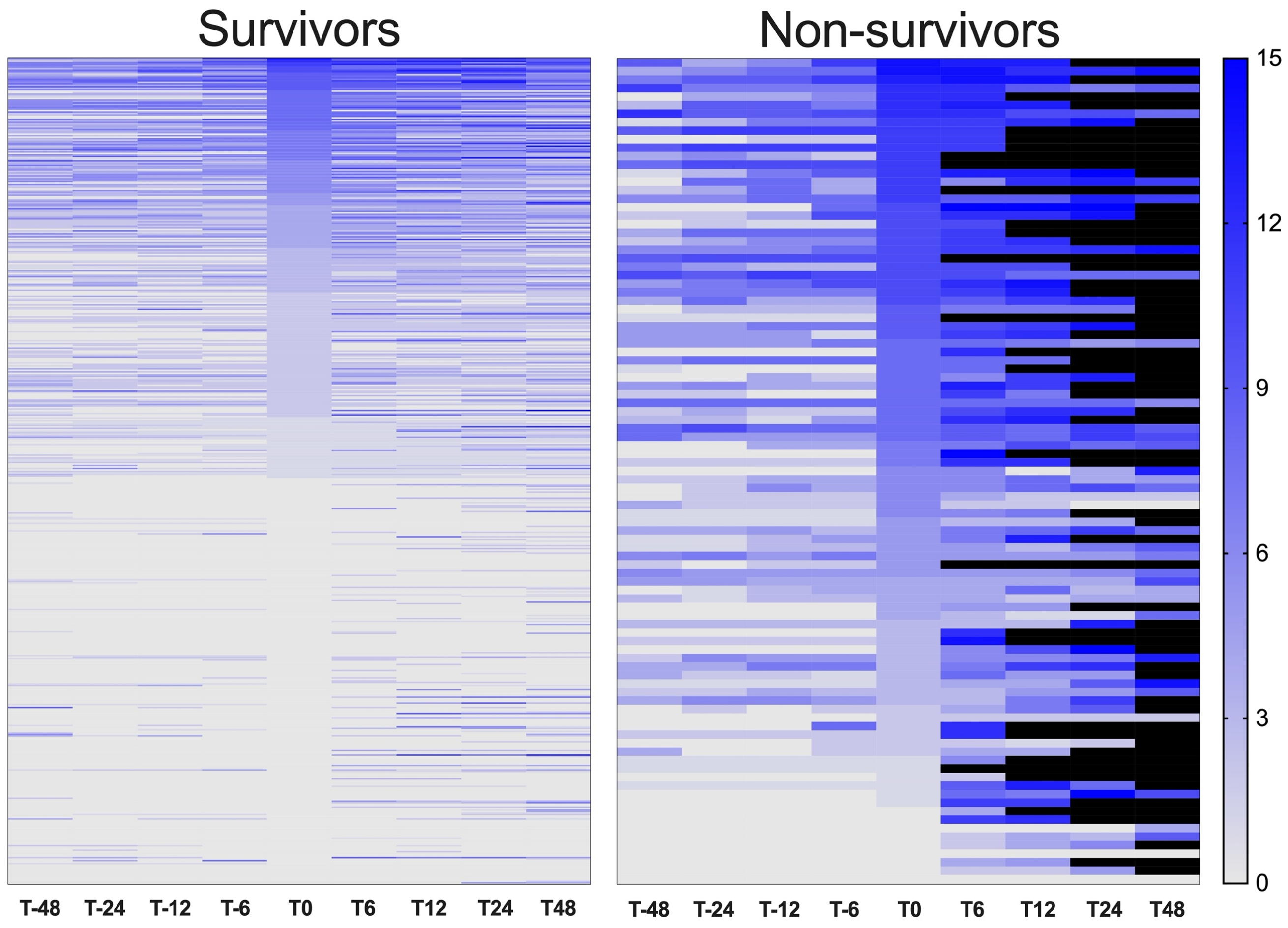

© 2021 Fleiss N et al. *JAMA Network Open.*

eFigure 7. Temporal progression of individual patient nSOFA$^{\Delta}$ dynamics among survivors and non-survivors. All patient values (survivors and non-survivors) are shown. Each row in the heat map represents a single patient. Each column represents a single time interval. The cell color and intensity reflects the nSOFA$^{\Delta}$ over the indicated time interval (legend) with no change indicated by white, a decreasing nSOFA in blue, and an increasing nSOFA in red.

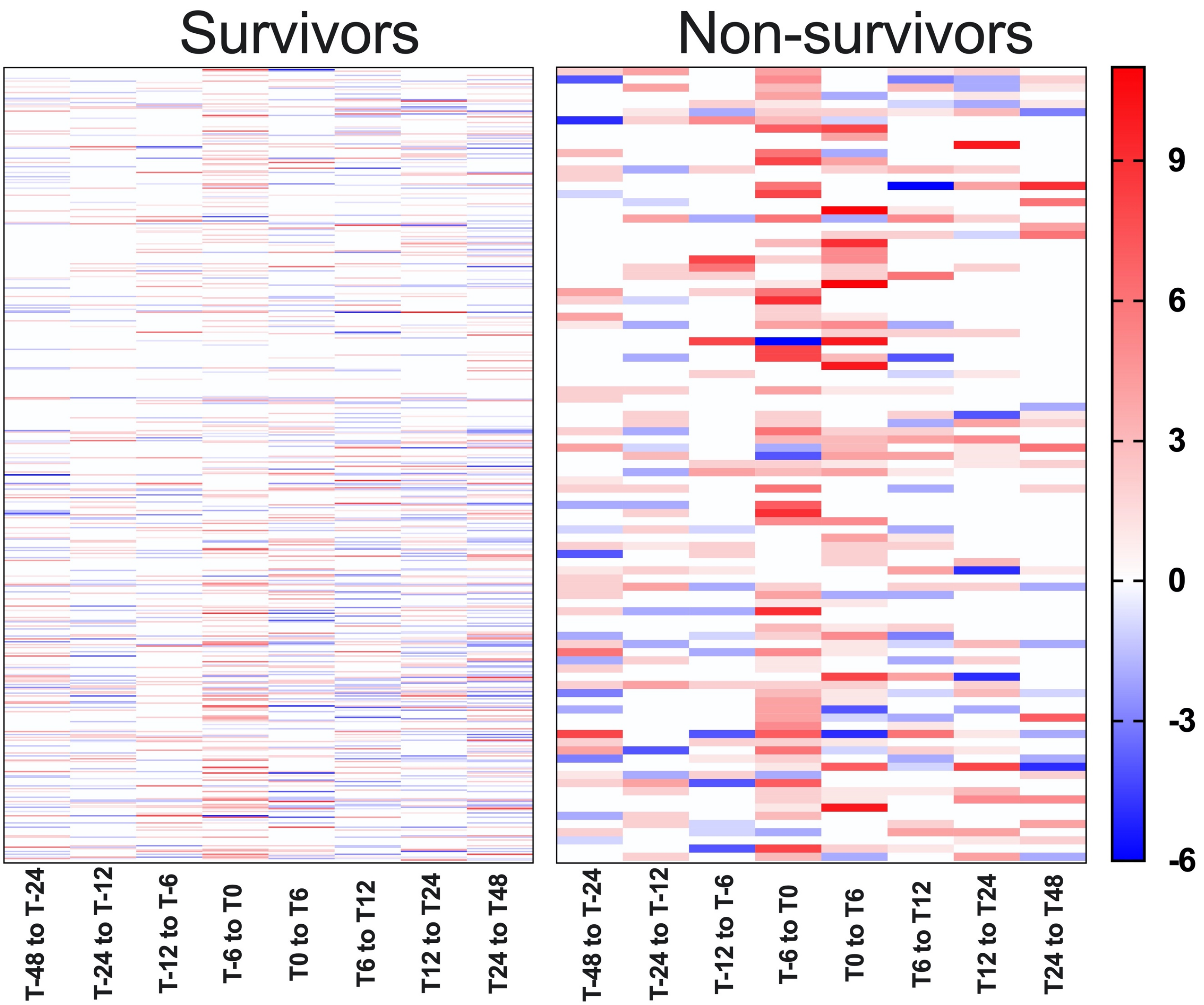

© 2021 Fleiss N et al. *JAMA Network Open.*

eFigure 8. Individual patient-level organ dysfunction dynamics among <25 weeks GA survivors and non-survivors. All patient values (survivors and non-survivors) are shown. Violin plots represent group medians at each time point. Error bars represent interquartile ranges (IQR). **A.** Pre-evaluation nSOFA$^{\Delta}$ was most pronounced among non-survivors between T-48 and T0 [median 3 (IQR: 0, 6), T-24 and T0 (3 (0, 5)), and T-6 and T0 (2 (0, 4); all p<0.001 by Mann-Whitney test]. **B.** nSOFA$^{\Delta}$ at post-evaluation time points. **C**. Peri-evaluation nSOFA$^{\Delta}$ differences increased proportionally to the length of time interval measured (T-6 to T6: median 3 (IQR: 1, 7); T-12 to T12: 4 (1, 7) ; T-24 to T24: 5 (2, 8); and T-48 to T48: 7 (3, 10). For patients that died prior to a time point, the most recent nSOFA score was used for calculations. * designates p-value <0.001. ** designates p-value <0.05.

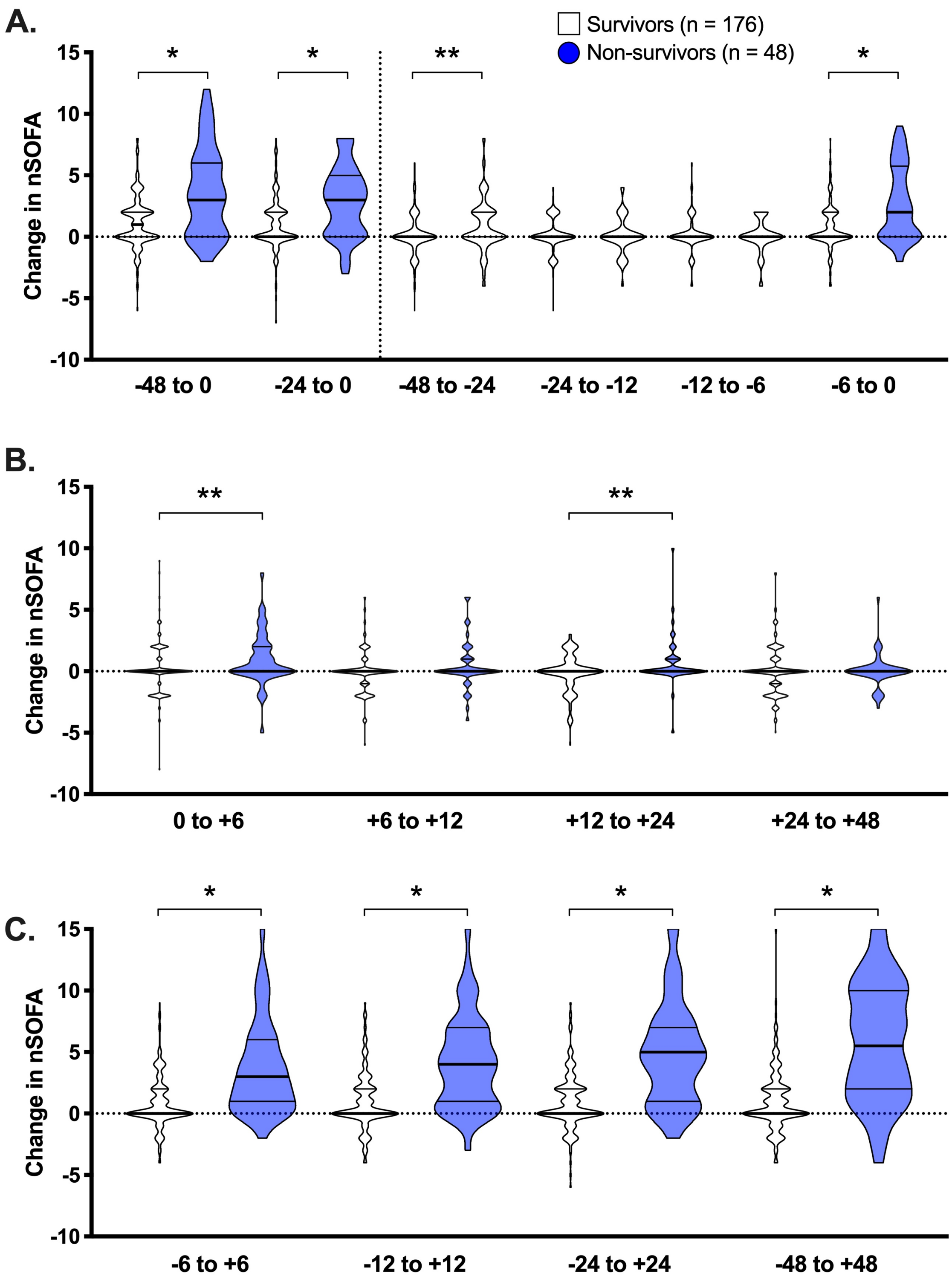

© 2021 Fleiss N et al. *JAMA Network Open.*

eFigure 9. Prevalence and temporal progression of organ dysfunction between survivors and non-survivors. Chord diagrams show each time point (**A.** T-48, **B.** T-24, **C.** T-12, **D.** T-6, **E.** T0, **F.** T6, **G.** T12, **H.** T24, **I.** T48). Ribbons connect patient groups (survivors, non-survivors) to organ systems [respiratory (RESP), cardiovascular (CV), hematologic (HEME)]. Ribbon thickness represents the percentage of patients in each category. Colored ribbons represent the presence of organ dysfunction ("+"; nSOFA > 0); grey ribbons within each component represent the absence of organ dysfunction ("-"; nSOFA = 0). The size of the outer band (yellow = survivors, black = non-survivors, and representative organ systems) is standardized and not representative of the number of patients.

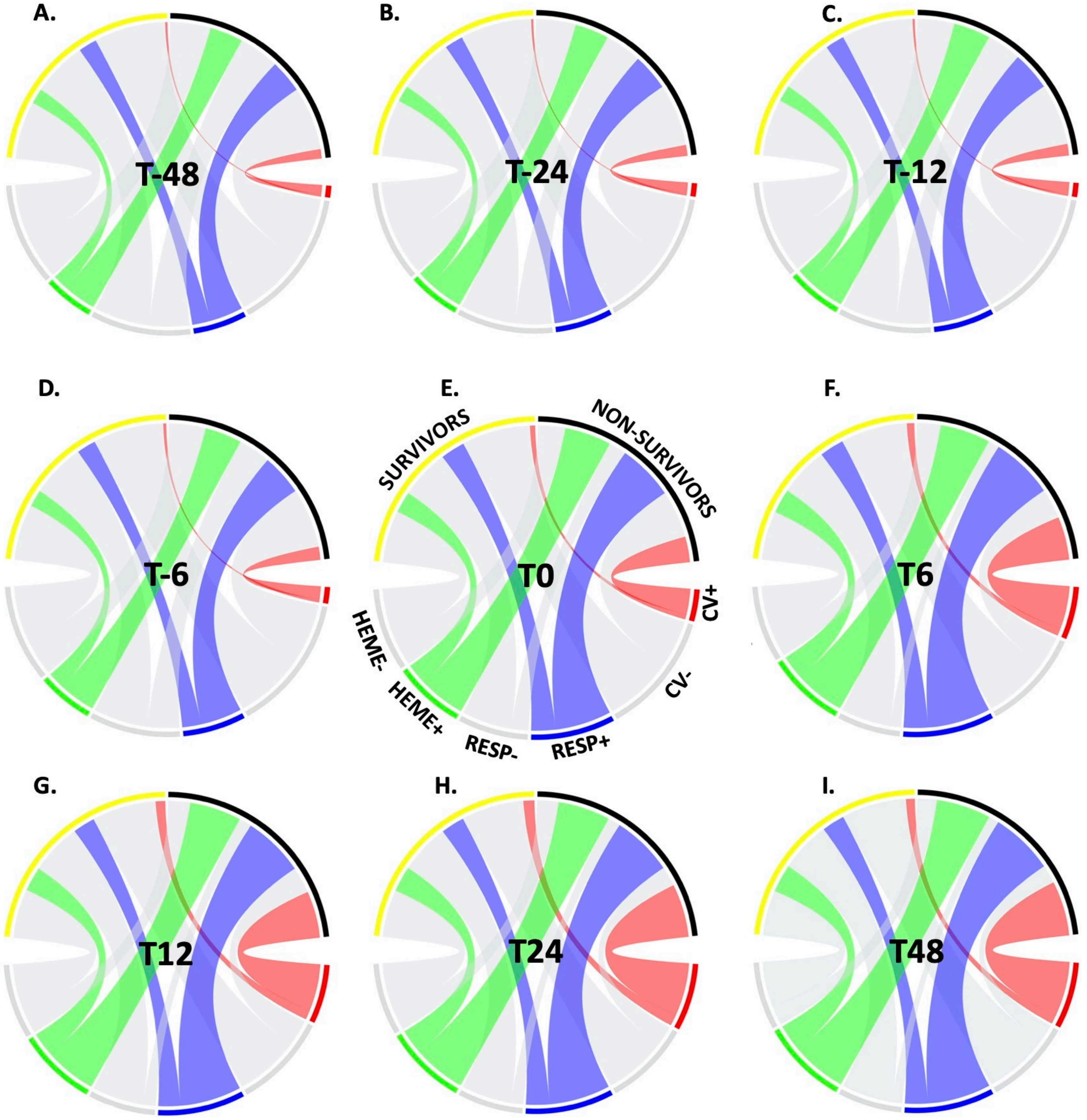

© 2021 Fleiss N et al. *JAMA Network Open.*

eFigure 10. nSOFA scores for females and males. Violin plots represent group medians at each time point. Error bars represent interquartile ranges (IQR). **A.** Comparison of nSOFA scores among females. **B.** Comparison of nSOFA scores among males. **C.** Area under receiver operating characteristics curves for the nSOFA to predict mortality in female infants at T0, T6, and T12 time points (all p<0.001). **D.** Area under receiver operating characteristics curves for the nSOFA to predict mortality in male infants at T0, T6, and T12 time points (all p<0.001). **E.** Comparison of nSOFA scores among female and male survivors. **F.** Comparison of nSOFA scores among female and male non-survivors. Comparisons by Mann-Whitney.

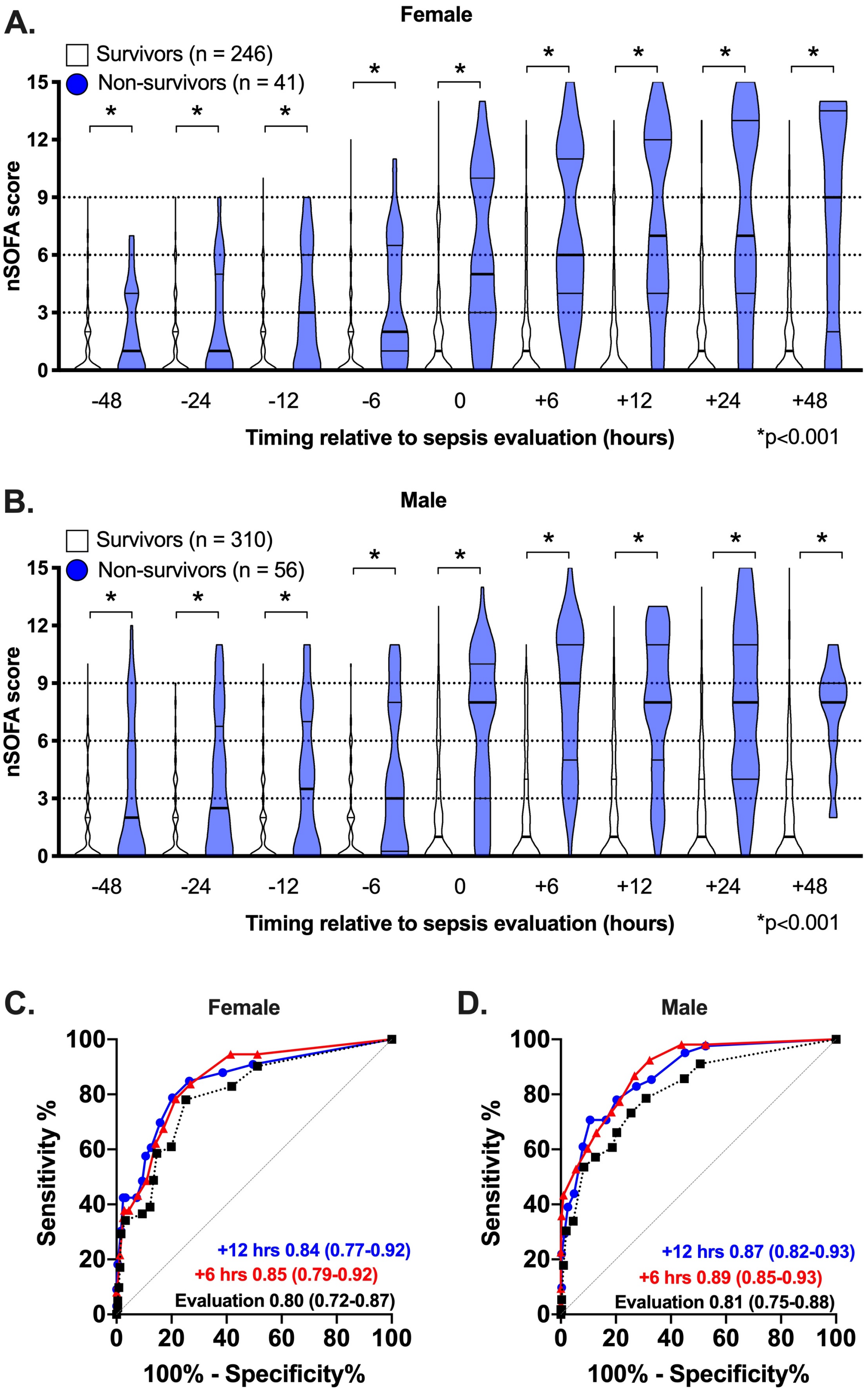

© 2021 Fleiss N et al. *JAMA Network Open.*

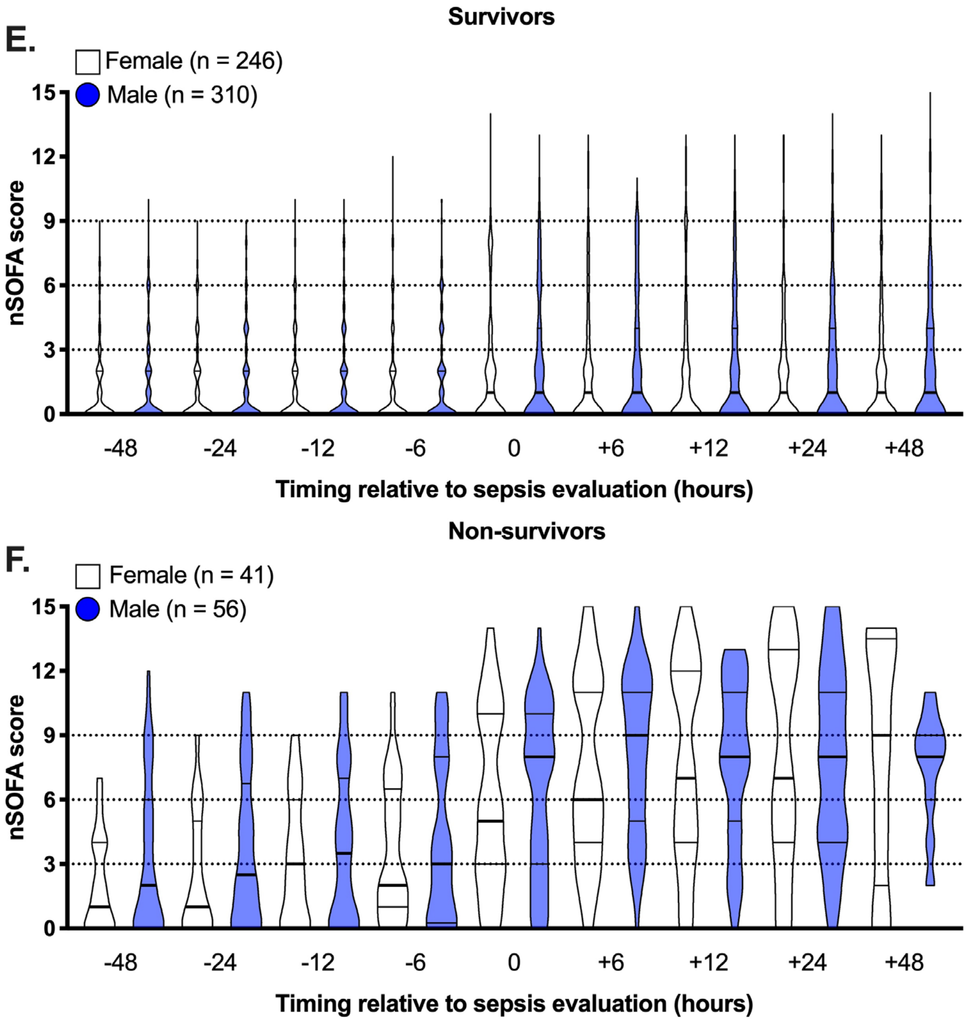

© 2021 Fleiss N et al. *JAMA Network Open.*

eFigure 11. nSOFA scores by pathogen classification. Violin plots represent group medians at each time point. Error bars represent interquartile ranges (IQR). **A.** Comparison of nSOFA scores among PATIENTS with gram-positive bacteremia. **B.** Comparison of nSOFA scores among PATIENTS with gram-negative bacteremia. **C.** Area under receiver operating characteristics curves for the nSOFA to predict mortality in infants with gram-positive bacteremia at T0, T6, and T12 time points (all p<0.001). **D.** Area under receiver operating characteristics curves for the nSOFA to predict mortality in infants with gram-negative bacteremia at T0, T6, and T12 time points (all p<0.001). **E.** Comparison of nSOFA scores among survivors with gram-positive or gram-negative bacteremia. **F.** Comparison of nSOFA scores among non-survivors with gram-positive or gram-negative bacteremia. Comparisons by Mann-Whitney. ** designates p-value <0.05.

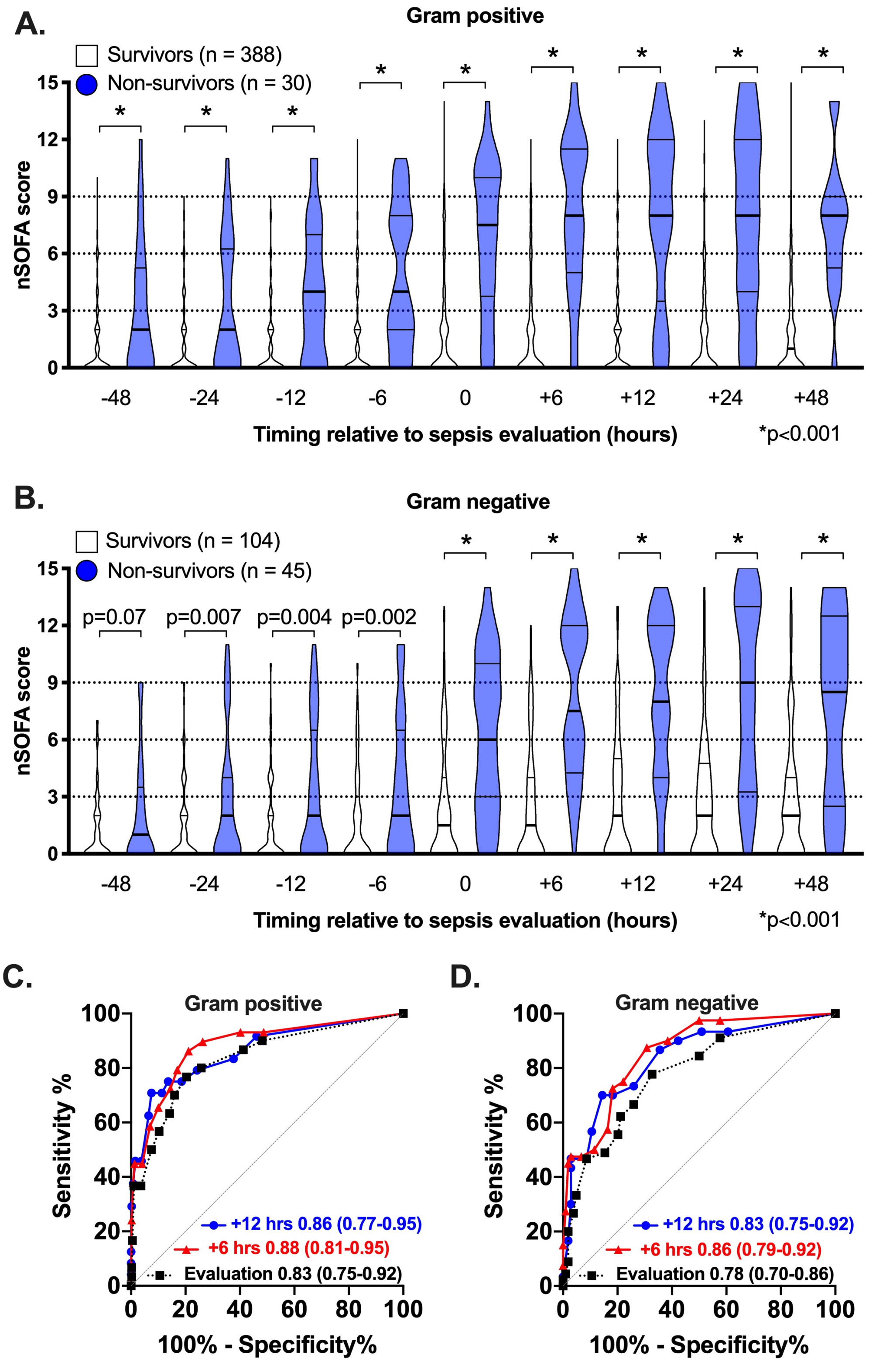

© 2021 Fleiss N et al. *JAMA Network Open.*

E.

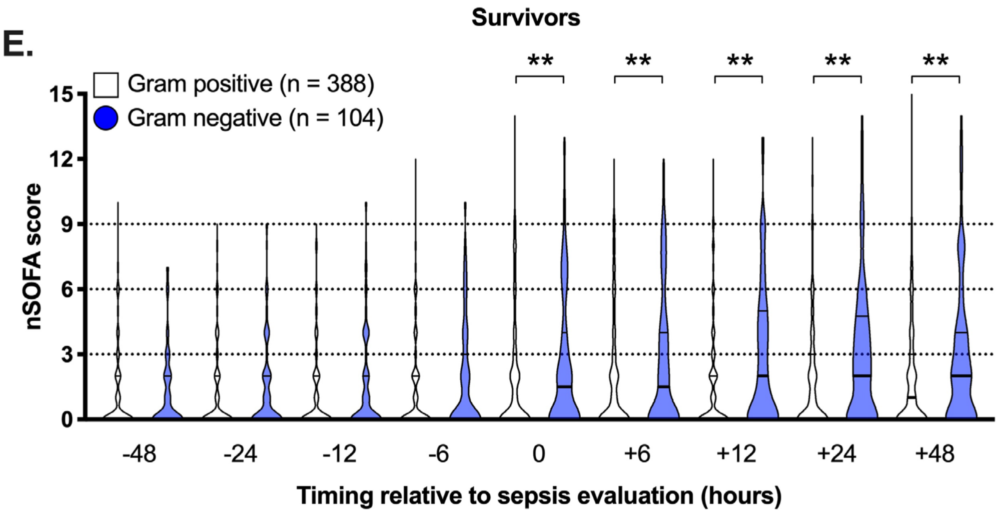

F.

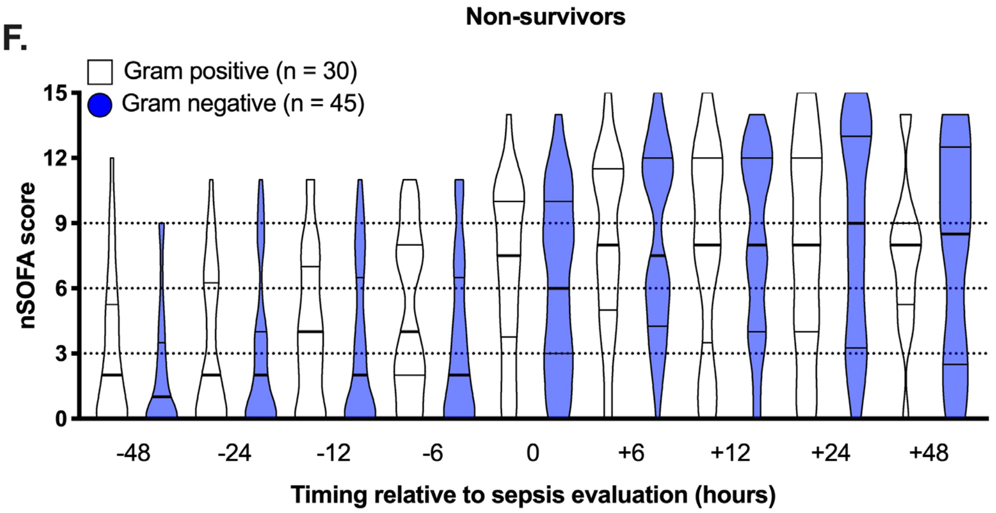

© 2021 Fleiss N et al. *JAMA Network Open.*

eFigure 12. nSOFA scores among patients with surgical peritonitis and negative blood cultures. **A.** Violin plots represent group medians at each time point. Error bars represent interquartile ranges (IQR). **B.** Area under receiver operating characteristics curve (AUROC) for death shown for T0 (squares), T6 (triangles), and T12 (circles) time points. All AUROCs with p<0.001. Comparisons by Mann-Whitney. * designates p-value <0.001. ** designates p-value <0.05.

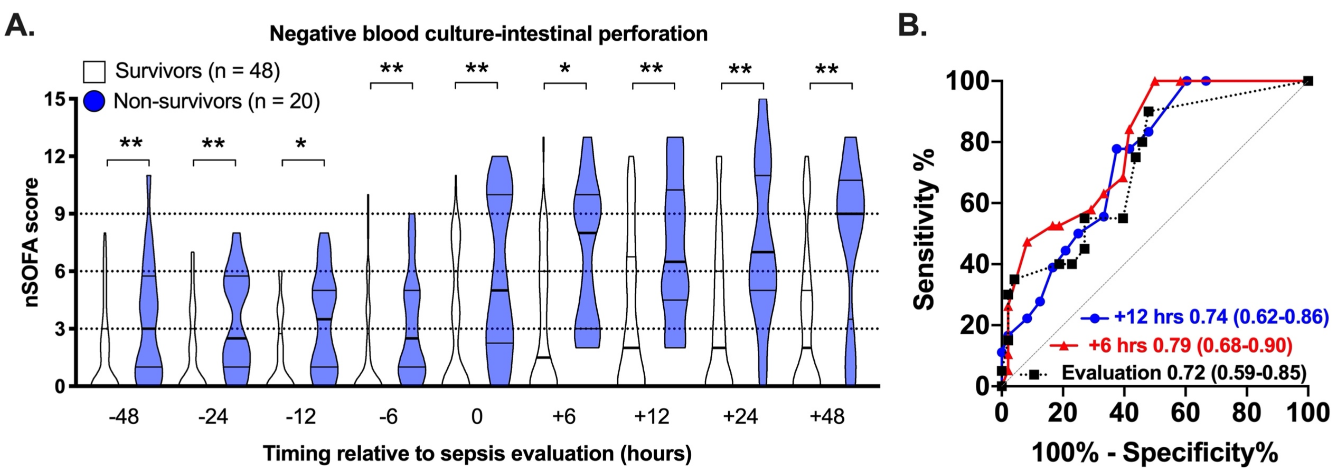

© 2021 Fleiss N et al. *JAMA Network Open.*

eAppendix. Neonatal Sequential Organ Failure Assessment (nSOFA)

nSOFA Background

One of the primary charges of the group that developed *The Third International Consensus Definitions for Sepsis and Septic Shock (Sepsis-3)* was to differentiate sepsis from uncomplicated infection.[1] As the authors of the Sepsis-3 guidelines stated:

1. "*Sepsis involves organ dysfunction, indicating a pathobiology more complex than infection plus an accompanying inflammatory response alone*".
2. "*For clinical operationalization, organ dysfunction can be represented by an increase in the Sequential [Sepsis-related] Organ Failure Assessment (SOFA) score of 2 points or more, which is associated with an in-hospital mortality greater than 10%*".
3. *"The SOFA score is not intended to be used as a tool for patient management but as a means to clinically characterize a septic patient."*

Thus, the SOFA score, and the nSOFA score are *not* used to determine initiation, continuation, or discontinuation of antibiotics, but rather to differentiate infection from sepsis by measuring the risk of death. In adults, the prerequisite to a diagnosis of sepsis supported by the SOFA score is "*suspected infection*"; evidenced by the clinical decision to send blood/body fluids for culture and start antibiotics.[1] For the retrospective, multi-center arm of this study to determine generalizability of the nSOFA to predict mortality with late-onset infection, the nSOFA was studied in patients with definitive infection (bacteremia or surgical peritonitis).

nSOFA components

The present study aimed to determine the generalizability of the nSOFA score. The nSOFA components and scoring paradigms were modeled after the adult SOFA and the pediatric SOFA.[1-3] As with the SOFA and pSOFA, nSOFA components and cutoffs aim to identify and quantify the severity the organ dysfunction that is associated with infection-related mortality. The derivation and single-center validation of the score has been previously described.[2, 4] The nSOFA utilizes only objective and clinical standard-of-care data to provide a serial measure of organ dysfunction that predicts mortality risk among preterm infants with late-onset infection. Importantly, the scoring designations within each system are not arranged as a reflection of the timing, sequence, or appropriateness of the interventions. In the studies that informed the development of the nSOFA[4], patients that died with late-onset bacteremia showed nearly universal receipt of intubation and mechanical ventilation as well as a doubling in oxygen requirement. The high prevalence of respiratory dysfunction associated with death compared to the prevalence of other organ dysfunctions justifies a greater weight in the score. The use of inotropic and vasoactive medications is associated with a high risk of mortality during infection.[5] However, we found nearly 60% of those that died with late-onset bacteremia never received inotropic or vasoactive medications.[4] To ensure generalizability, the nSOFA score uses the most recent platelet count *available to the clinician*. Multiple measurements of platelets would be considered outside clinical practice and thus the results obtained using that approach would not be generalizable. As a result, a single platelet measurement, sent at the discretion of the clinician, may be used in the calculation of more than one nSOFA score. There were no instances for any patient in this study where a platelet measure was unavailable.

Systems not included in nSOFA

The adult SOFA and pSOFA include component scores from six systems: respiration, coagulation, cardiovascular, liver, renal, and central nervous system. The nSOFA includes assessments of respiratory, cardiovascular, and hematologic dysfunction, but does not include assessments of renal, hepatic, or central nervous system dysfunction. We argue these exclusions are justified concurrently by the following rationale:

1) Total bilirubin is the component used to measure liver dysfunction in the SOFA. Bilirubin measures, while potentially informative in critically-ill children and adults, are complicated by protracted physiologic jaundice in preterm neonates.
2) In contrast to adults and children, where continuous renal replacement therapy (CRRT) is used to mitigate renal failure, the use of CRRT in neonates is extremely rare in the absence of severe congenital kidney disease. Furthermore, the current definition of acute kidney injury in neonates is an increase in serum creatinine of $\geq$0.3 mg/dL or $\geq$50% from the previous lowest value, or a urinary output of less than 1 mL/kg per hour on post-natal days 2-7.[6] Outside of the post-natal age restrictions that would decrease generalizability past the first week of life, both creatinine and urine output have multiple co-covariates including post-natal age, fluid intake, presence of hypotension, concurrent use of diuretics, systemic glucocorticoids, antidiuretic hormone concentrations that may occur with central nervous system injury, gestational age, and maternal creatinine.
3) Objective central nervous system *functional* assessments applicable to preterm infants are not well-established and thus not commonly used in the NICU population. Descriptive characteristics commonly used such as "*lethargy"* are highly-subjective. Other assessments including tone and responsiveness to touch or pain are affected by gestational age and the presence of sedation.

Based on these many limitations, central nervous system, renal, and hepatic system assessments were not included in the present iteration of the nSOFA. Additionally, neither the total white blood cell count nor the absolute neutrophil count are included in the adult SOFA or the pSOFA.[1, 3]

eReferences

1. Singer M, Deutschman CS, Seymour CW, et al. The Third International Consensus Definitions for Sepsis and Septic Shock (Sepsis-3). JAMA. 2016;315(8):801-810.
2. Wynn JL, Polin RA. A neonatal sequential organ failure assessment score predicts mortality to late-onset sepsis in preterm very low birth weight infants. Pediatr Res. 2020;88(1):85-90.
3. Matics TJ, Sanchez-Pinto LN. Adaptation and Validation of a Pediatric Sequential Organ Failure Assessment Score and Evaluation of the Sepsis-3 Definitions in Critically Ill Children. JAMA pediatrics. 2017;171(10):e172352.
4. Wynn JL, Kelly MS, Benjamin DK, et al. Timing of Multiorgan Dysfunction among Hospitalized Infants with Fatal Fulminant Sepsis. Am J Perinatol. 2017;34(7):633-639.
5. Kermorvant-Duchemin E, Laborie S, Rabilloud M, Lapillonne A, Claris O. Outcome and prognostic factors in neonates with septic shock. Pediatr Crit Care Med. 2008;9(2):186-191.
6. Jetton JG, Boohaker LJ, Sethi SK, et al. Incidence and outcomes of neonatal acute kidney injury (AWAKEN): a multicentre, multinational, observational cohort study. Lancet Child Adolesc Health. 2017;1(3):184-194.

© 2021 Fleiss N et al. *JAMA Network Open.*
